# Intelligent computing technique based supervised learning for squeezing flow model

**DOI:** 10.1038/s41598-021-99108-z

**Published:** 2021-10-01

**Authors:** Maryam Mabrook Almalki, Eman Salem Alaidarous, Dalal Adnan Maturi, Muhammad Asif Zahoor Raja, Muhammad Shoaib

**Affiliations:** 1 Department of Mathematics, Faculty of Science, Umm Al-Qura University, Makkah, 24211 Saudi Arabia; 2 Department of Mathematics, Faculty of Science, King Abdulaziz University, Jeddah, 21589 Saudi Arabia; 3 Future Technology Research Center, National Yunlin University of Science and Technology, Douliu, 64002 Taiwan; 4 Department of Mathematics, COMSATS University Islamabad, Attock Campus, Attock, 43600 Pakistan

**Keywords:** Materials science, Physics

## Abstract

In this study, the unsteady squeezing flow between circular parallel plates (USF-CPP) is investigated through the intelligent computing paradigm of Levenberg–Marquard backpropagation neural networks (LMBNN). Similarity transformation introduces the fluidic system of the governing partial differential equations into nonlinear ordinary differential equations. A dataset is generated based on squeezing fluid flow system USF-CPP for the LMBNN through the Runge–Kutta method by the suitable variations of Reynolds number and volume flow rate. To attain approximation solutions for USF-CPP to different scenarios and cases of LMBNN, the operations of training, testing, and validation are prepared and then the outcomes are compared with the reference data set to ensure the suggested model’s accuracy. The output of LMBNN is discussed by the mean square error, dynamics of state transition, analysis of error histograms, and regression illustrations.

## Introduction

In fluid dynamics, several areas inspire the researchers to further study and explore applicability and analysis. The flow of squeezing between two parallel circular walls is one of them because of its many valuable and varied applications in our current life reality. The primary vital application is the heart, where it pumps blood to the entire body through pressure. It also has industrial applications and engineering such that injection molding and polymer processing. Stefan^1^ publication of a classical study of squeezing flow through the use of lubrication to generate a homogeneous compression provides an aspect to study squeezing flow system. This study is inspired by a series of studies on squeezing flow system investigated by many researchers. Ahmed et al.^2^ studied the unsteady squeezing flow considering the viscosity mainly affected by the temperature by applying the killer box method. Çelik et al.^3^ investigated the influence of heat transfer and velocity on squeezing flow by the Gegenbauer Wavelet Collocation Method. Sobamowo et al.^4^ used both methods of differential transformation and variation of parameters to study the effect of a magnetic field on Casson nanofluid’s squeezing flow through a porous medium. Çelik^5^ studied the effect of viscosity on squeezing flow in a magnetic field for a specific type of fluid known as Copper-water and Copper-kerosene. Noor et al.^6^ discussed the impact of Cattaneo–Christov heat and mass fluxes on nanofluid’s squeezing flow. Usman et al.^7^ introduced new improvements to the wavelets method that helped to analyze the unsteady flow of nanofluid between two disks. Thumma et al.^8^ examined the influence of convection on the flow problem of electromagnetohydrodynamic radiative between two circular plates. Some other recent studies that have addressed squeezing flow can be seen in the literature^9–14^.

In the previous research, squeezing flow has been studied using different numerical methods, but stochastic numerical computing that is dealing with artificial intelligence is utilized to analyze the fluidic systems recently.

The accurate results provided by stochastic numerical computing have been employed to provide new research in various fields such as fluid mechanics^15–17^, biological research^18,19^, business and finance systems^20,21^, models of Panto-graph delay differential systems^22–24^, plasma science^25^, thermodynamics^26^, magneto-hydrodynamics^27^, solid conductive materials^28^, atomic physics^29^ and other researches of interest.It is worth noting that artificial intelligence is also able to keep pace with modern problems that are emerging in the world in various fields, such as Covid 19^30,31^.

In this study, the system of (USF-CPP) is performed by an intelligent computing paradigm of Levenberg-Marquard backpropagation neural networks (LMBNN). The research proceeds in several steps that can be summarized as follows

- Levenberg-Marquard backpropagation neural networks (LMBNN) is developed to discuss the impact of different scenarios connected with the squeezing flow system (USF-CPP).
- The governing flow system (USF-CPP) based on partial differential equations (PDEs) is transformed into differential equations (ODEs) for better applicability of networks (LMBNN).
- Runge-Kutta method is used to generate a dataset for the USF-CPP problem, which is finally prepared for neural network infrastructure, i.e., LMBNN by variation of Reynolds number and volume flow rate.
- LMBNN processes that are testing, training, and validation applied on system presenting the squeezing flow model USF-CPP for various scenarios and cases.
- The mean square error discusses the results of LMBNN, dynamics of state transition, analysis of error histograms, and regression illustrations.

**Figure 1:**
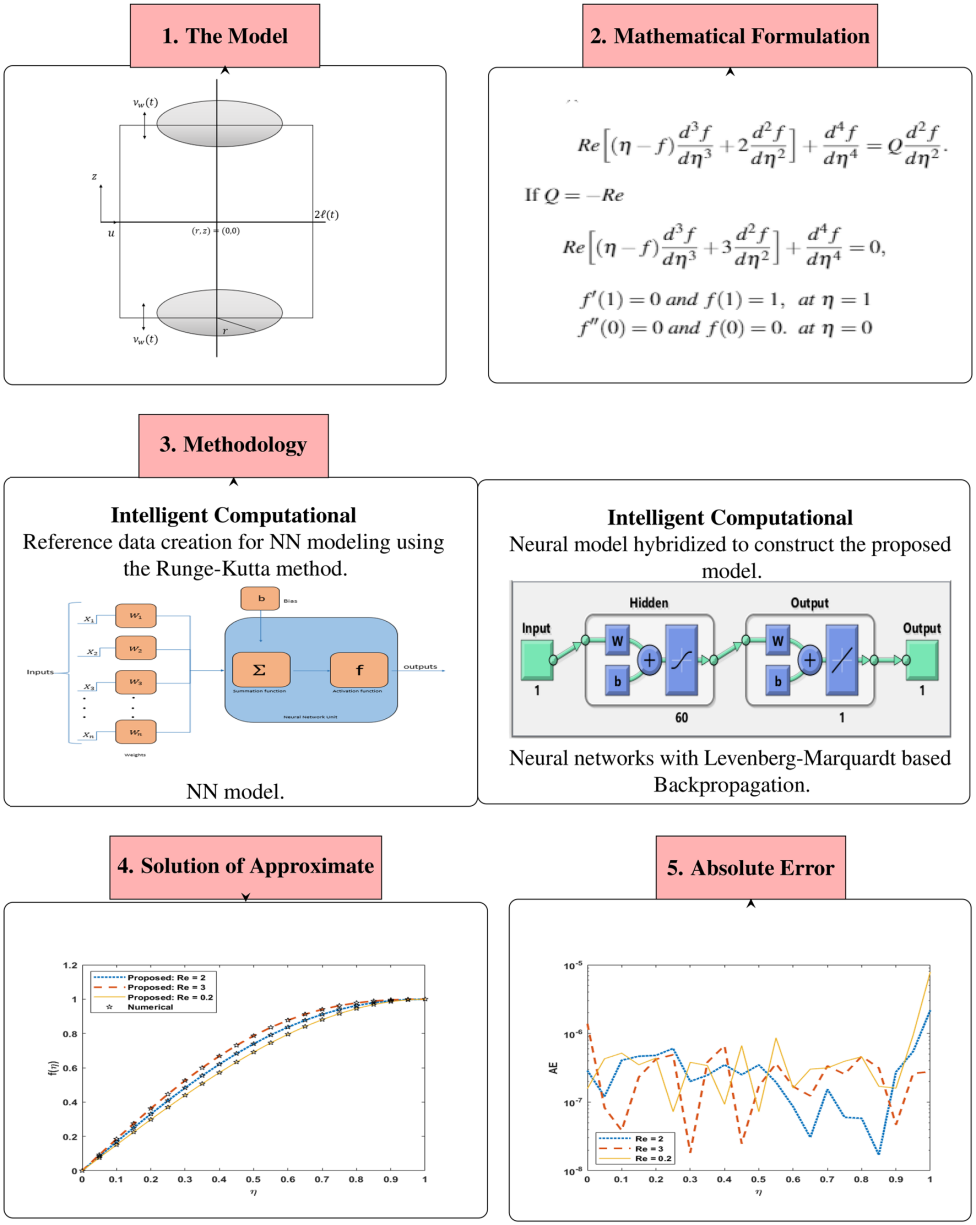
The diagram of the proposed LMBNN for solving the USF-CPP model.

The workflow overview of solving USF-CPP with the proposed model LMBNN is presented in (See Fig. 1). The Mathematical formulation of the USF-CPP model exposure in “Solution methodology” section. The present model solution Procedure has been displayed in “Results and discussion” section. The accuracy of the output, the proposed LMBNN, is showing in “Conclusions” section. The conclusion of the research is given in the last section.

## Mathematical formulation

**Figure 2:**
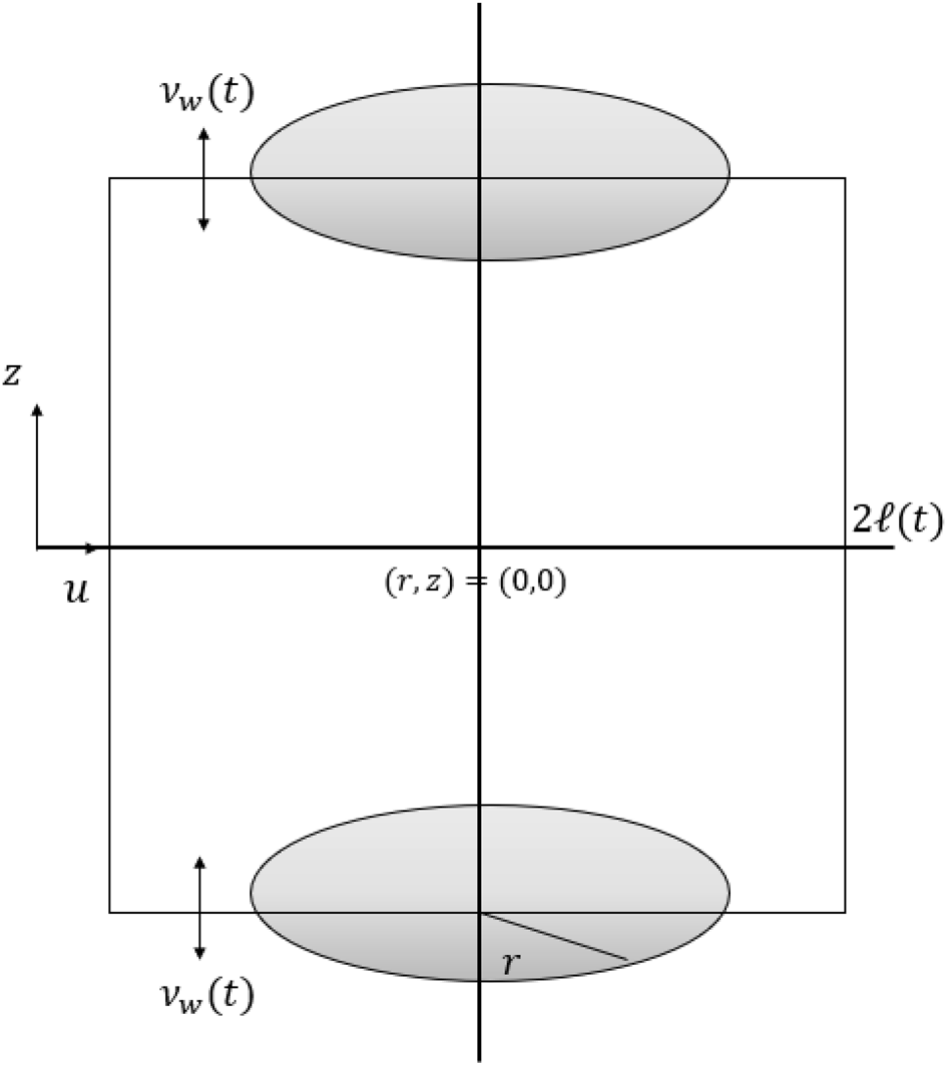
System scheme of USF-CPP.

The geometry of the squeezing flow of an incompressible two-dimensional viscous fluid between two parallel plates shown in (See Fig. 2). The distance between the two circular plates at any time t is $2\ell (t)$. The speed at which the upper and lower plates move each other is *v*(*t*). Select the *r*-axis as the model’s central axis, and the *z*-axis is normal to it. For axisymmetric flow, assumed that the plates approach symmetrically with respect to *r*-axis.

The governing system^32^ become in form

1 $$\begin{aligned}&{\frac{1}{r} \partial _{r}(ru)+\partial _{z}w=0,} \end{aligned}$$

2 $$\begin{aligned}&{\rho (\partial _{t}u+u\partial _{r}u+w\partial _{z}u)=-\partial _{r} p+\mu \left( \nabla ^{2}u-\frac{u}{r^{2}}\right) ,} \end{aligned}$$

3 $$\begin{aligned}&{\rho (\partial _{t}w+u\partial _{r}w+w\partial _{z}w)=-\partial _{z} p+\mu \nabla ^{2}w,} \end{aligned}$$

where

4 $$\begin{aligned} \begin{aligned} \nabla ^{2}&=\partial ^{2}_{r^2}+\frac{1}{r}\partial _{r}+\partial ^{2}_{z^2},\\ \partial _{k}&=\frac{\partial }{\partial {k}}\ \ \ {\mathrm{and}} \ \ \partial ^{2}_{k^2}=\frac{\partial ^2}{\partial {k^2}}.\\ \end{aligned} \end{aligned}$$

Subject to the boundary conditions

5 $$\begin{aligned} \begin{aligned} u=0\ {\mathrm{and}} \ w=v_w(t),\ \ at \ \eta&=1\\ \partial _ {\eta }u=0\ {\mathrm{and}} \ w=0,\ \ at\ \eta&=0 \end{aligned} \end{aligned}$$

where $\eta =\frac{z}{\ell (t)}$, *u* radial velocity and *w* axial velocity.

To simplify the complex system of differential equations above and make it easier to find and analyze the results, we use similarity transformations and the following equation yields.

6 $$\begin{aligned} {Re\Big [(\eta -f) d^3_{\eta ^3}f+2d^2_{\eta ^2}f\Big ]+d^4_{\eta ^4}f=Qd^2_{\eta ^2}f,} \end{aligned}$$

where both *Re* and *Q* are constant.

The circular plates diverge when $Re\>0$, while converges towards each other when $Re<0$ and the squeezing flow are symmetrical with the velocity profiles, provided $\ell (t)\>0$. As well if $Q=-Re$ then Eq.(6) is reduced to

7 $$\begin{aligned} {Re\Big [(\eta -f)d^3_{\eta ^3}f+3d^2_{\eta ^2}f\Big ]+d^2_{\eta ^2}f=0,} \end{aligned}$$

where

8 $$\begin{aligned} \begin{aligned} d_{\eta }=\frac{d}{d{\eta }}\ \ \ {\mathrm{and}} \ \ d^{2}_{\eta ^2}=\frac{d^2}{d{\eta ^2}}. \end{aligned} \end{aligned}$$

With the following boundary conditions

9 $$\begin{aligned}&f^{\prime}(1)=0\ {\mathrm{and}} \ f(1)=1,\ \ at \ \eta =1\\&f^{\prime\prime}(0)=0\ {\mathrm{and}} \ f(0)=0.\ \ at \ \eta =0 \end{aligned}$$

## Solution methodology

**Figure 3:**
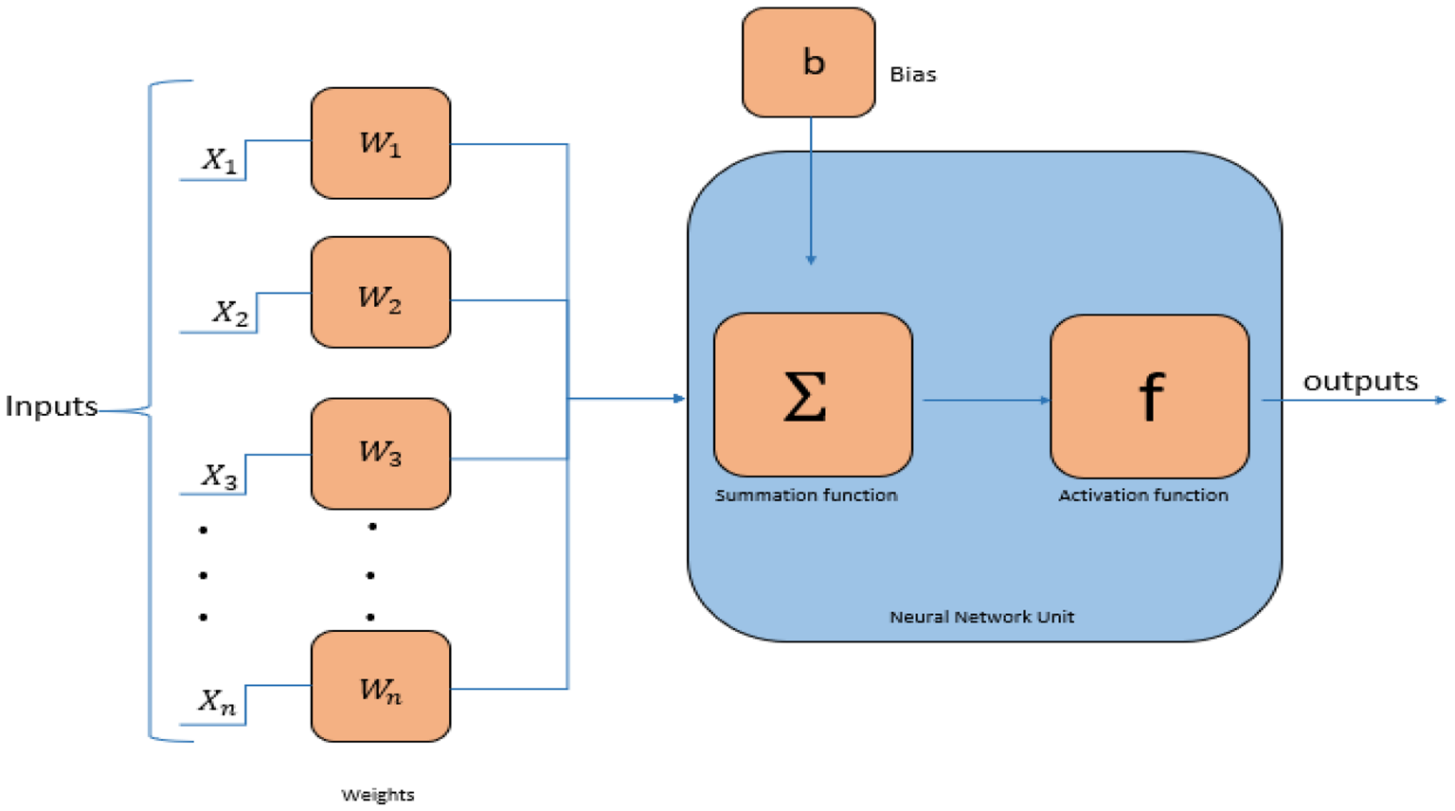
A single neural model structure.

The Levenberg Marquardt (LM) training technique is an efficient technique in the field of intelligent computing. It is designed to calculate the second-order training fast, and it requires that the output of the neural network operation is a single neuron (See Fig. 3).

**Figure 4:**
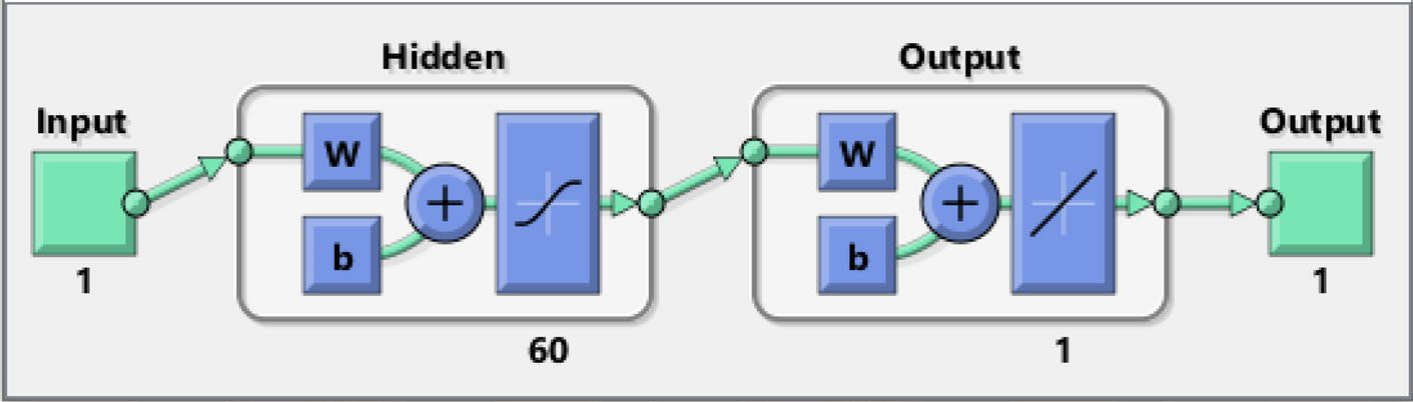
Installation of neural network.

Implement the Levenberg Marquardt technique in MATLAB based on using the command of the neural network toolbox “nftool” to fit the problem. The total data for LMBNN is 1001 found between 0 and 1 by setting 0.001 as steps, using the Runge-Kutta technique through the “NDSolve” built-in function for numerical solution in Mathematica. The dataset values for $f(\eta )$ were randomly used for each of the training, validation, and testing with $70\%$, $15\%$, $15\%$, respectively. For accurate results, select 60 as the number of neurons. The LMBNN is a computational model with Double neural network coats (See Fig. 4).

## Results and discussion

The numerical application based on LMBNN is presented here for the squeezing flow model obtained in Eqs. (6-9). The proposed LMBNN is implemented for six scenarios by variation of *Re*, *Q*, with three different cases for each scenarios, as shown in Table 1. Notice that the equation associated with value variation is used in each scenario.

**Table 1:** Scenarios and cases distribution for USF-CPP model.

Scenarios(S)	Cases	The physical parameters under study	
		Re	Q
(1)	1	2	–
$Q=-Re$	2	3	–
	3	0.2	–
(2)	1	0.2	–
$Q=-Re$	2	2	–
	3	3	–
(3)	1	$-$6	–
$Q=-Re$	2	$-$15	–
	3	$-$0.9	–
(4)	1	$-$6	–
$Q=-Re$	2	$-$15	–
	3	$-$0.9	–
(5)	1	1	0.2
$Q\ne -Re$	2	1	6
	3	1	15
(6)	1	1	0.2
$Q\ne -Re$	2	1	6
	3	1	15

**Figure 5:**
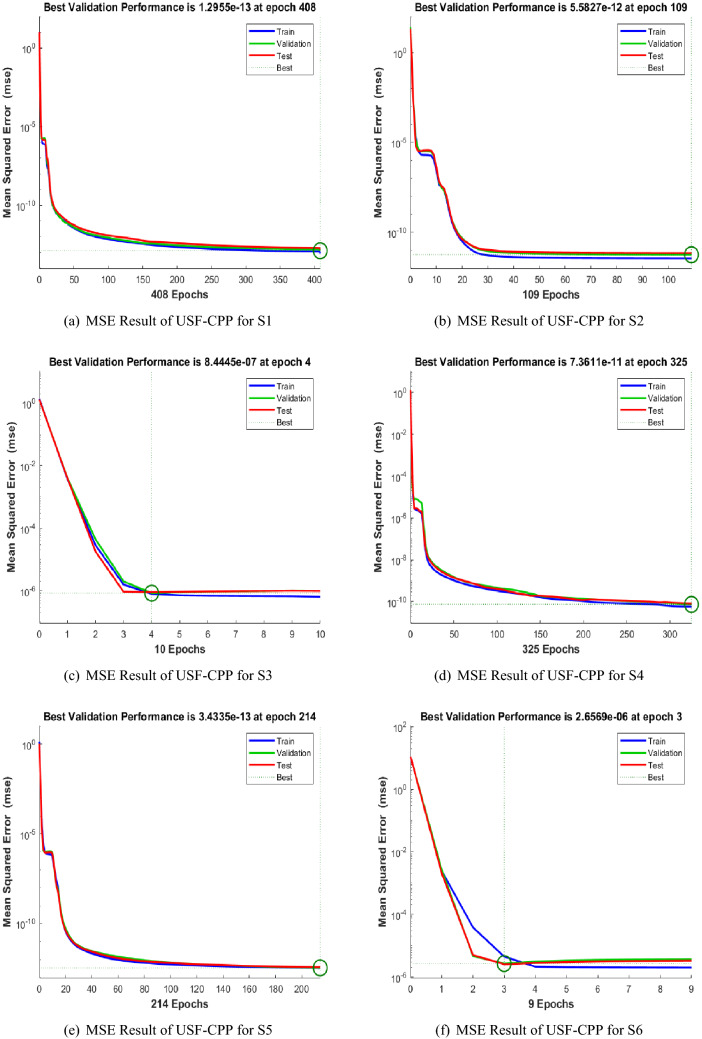
LMBNN Performance based on MSE for USF-CPP (Case 2).

**Figure 6:**
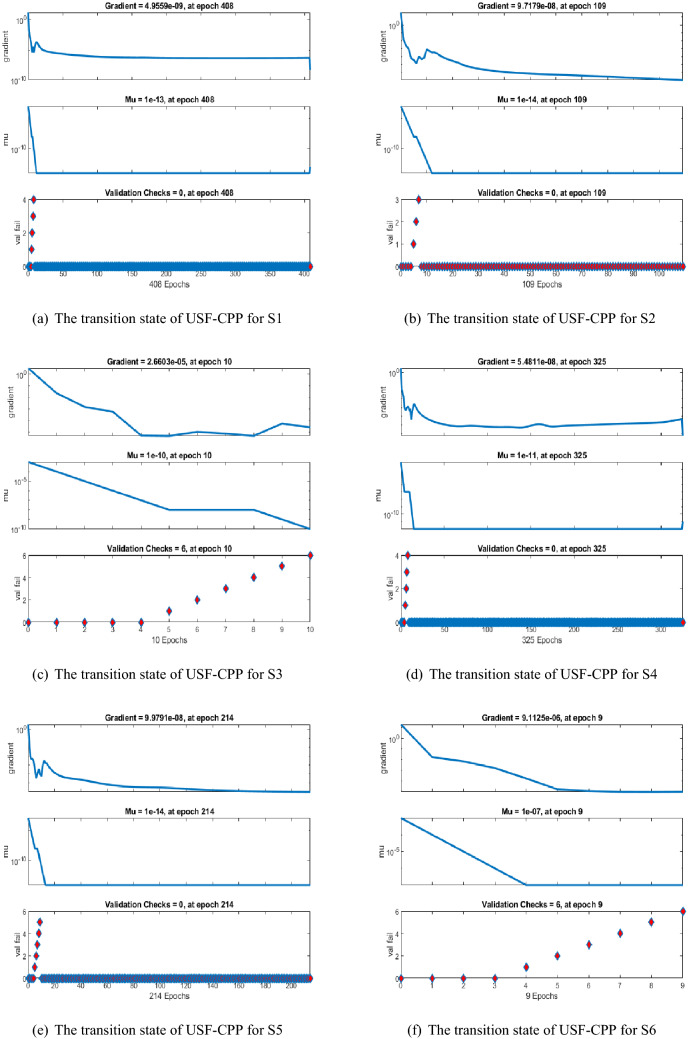
LMBNN Performance based on Gradient, Mu, and validation for USF-CPP (Case 2).

**Figure 7:**
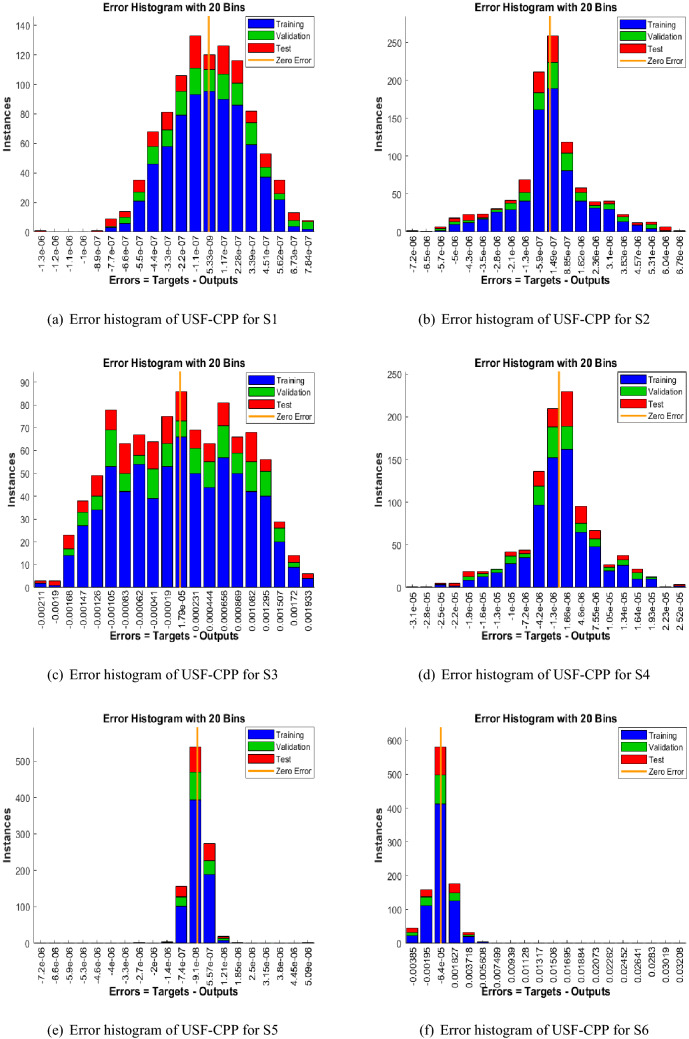
LMBNN studies based on error histogram for USF-CPP (Case 2).

**Figure 8:**
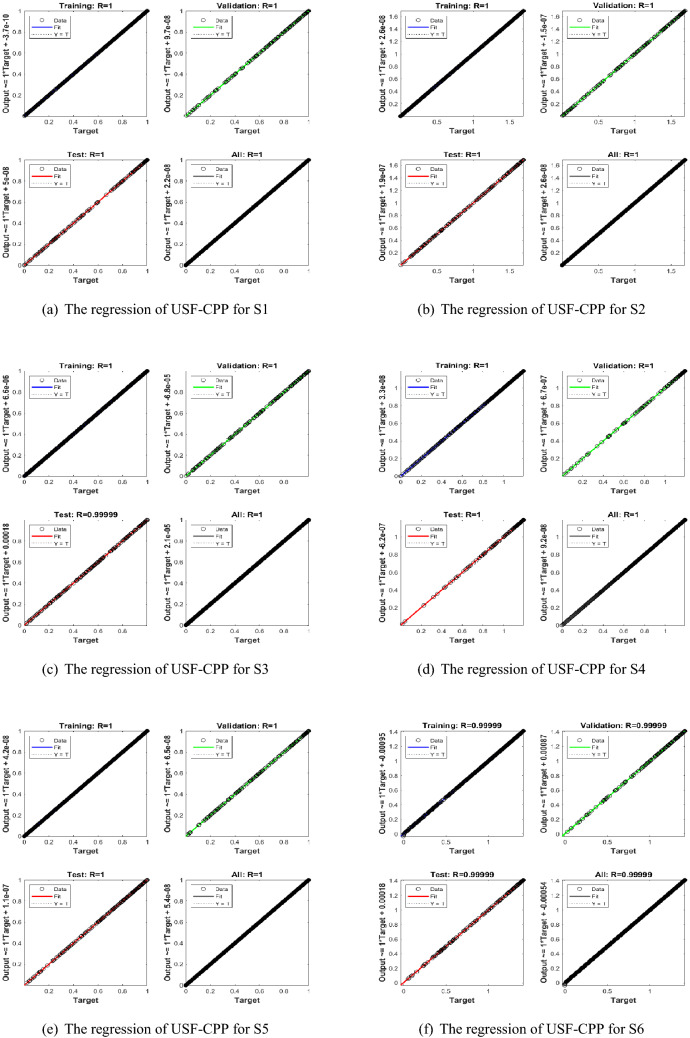
LMBNN studies based on regression for USF-CPP (Case 2).

**Figure 9:**
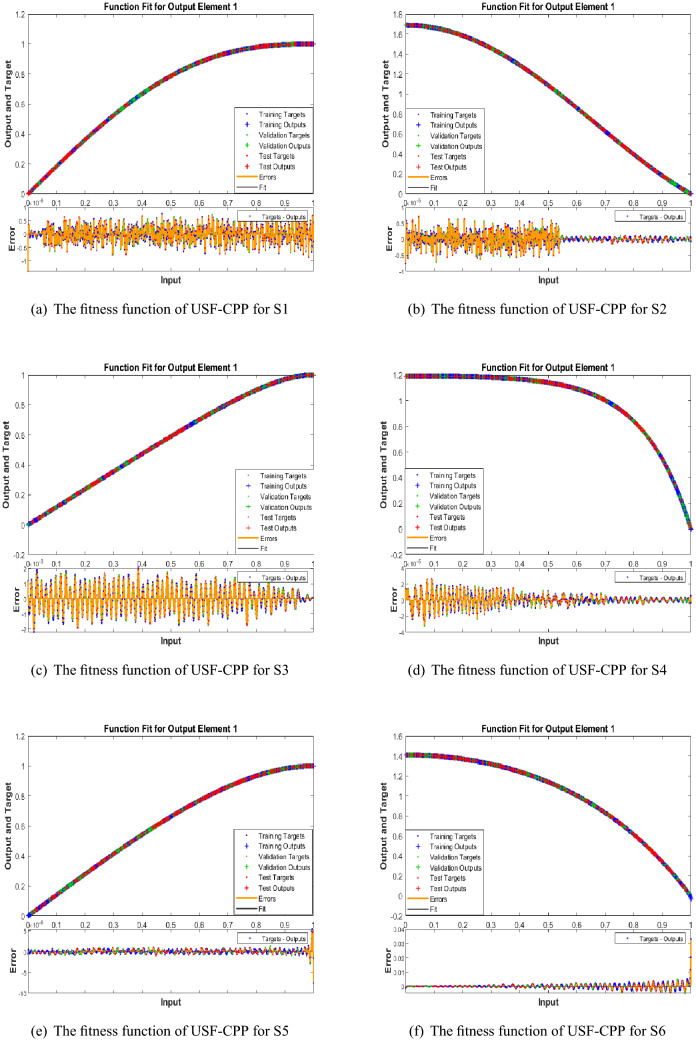
LMBNN analyses based on fitness function for USF-CPP (Case 2).

**Figure 10:**
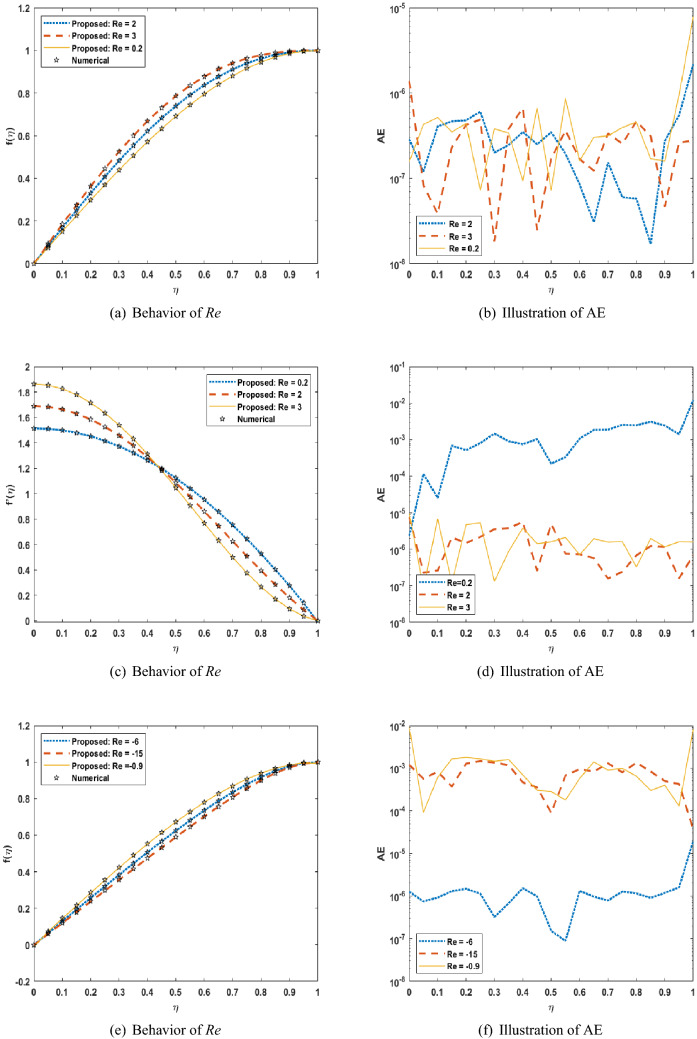
LMBNN Result and numerical reference results of USF-CPP for S1 to S3.

**Figure 11:**
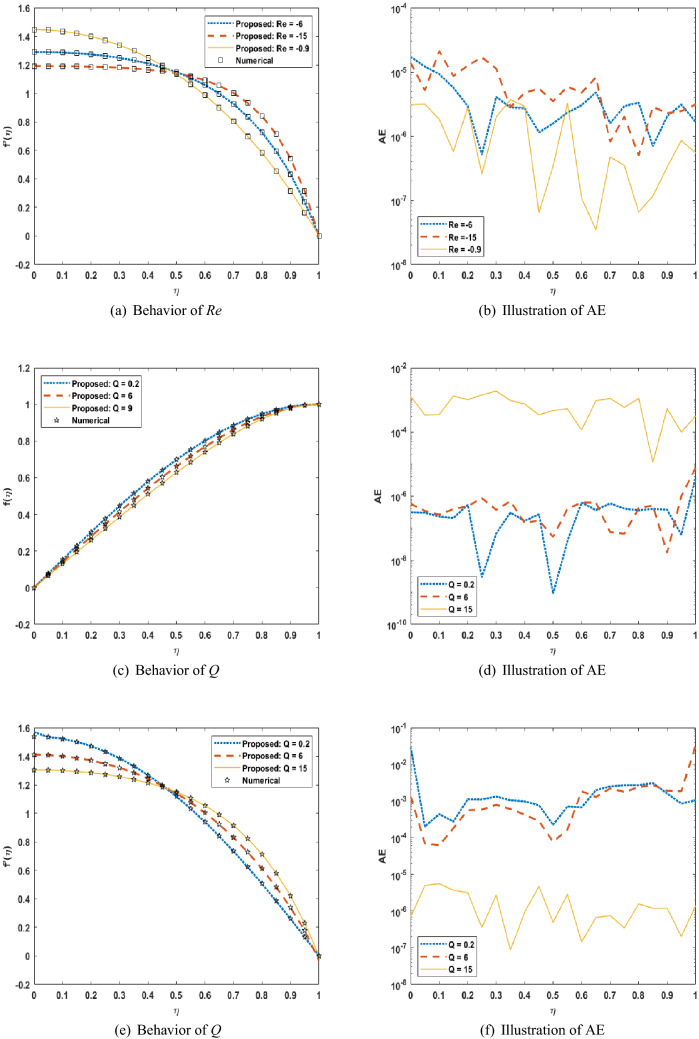
LMBNN Result and numerical reference results of USF-CPP for S4 to S6.

Figures 5, 6, 7 shows that performance, states, and error histograms for all six scenarios in case 2 for USF-CPP, respectively. Studies of regression are given (See Fig. 8). The fitting of solution respective six scenarios of case 2 is presented (See Fig. 9). Also, LMBNN outcomes are comparing with the standard outcomes (See Figs. 10, 11).

The mean squared error (MSE) for all three operations is given (See Fig. 5) to validate all different scenarios. Epochs performance clearly Check in 408, 109, 4, 325, 214, 3 while MSE is around ($10^{-12}$ to $10^{-13}$, $10^{-11}$ to $10^{-12}$, $10^{-06}$ to $10^{-07}$, $10^{-10}$ to $10^{-11}$, $10^{-12}$ to $10^{-13}$, $10^{-05}$ to $10^{-06}$) respectively (See Fig. 5).

The gradient of case 2 for all six scenarios respectively around ($4.95\times 10^{-09}$, $9.72\times 10^{-08}$, $2.66\times 10^{-05}$, $5.48\times 10^{-08}$, $9.98\times 10^{-08}$, $9.11\times 10^{-06}$) and the backpropagation measures is around ($10^{-13}, 10^{-14}, 10^{-10}, 10^{-11}, 10^{-14}, 10^{-07}$) (See Fig. 6). Analyze of the varition error histograms for differents points is presented (See Fig. 7). The zero axes along with the error box of reference for all six scenarios in case 2 is around ($5.33\times 10^{-09}$, $1.49\times 10^{-07}$, $1.79\times 10^{-05}$, $-1.3\times 10^{-06}$, $-9.1\times 10^{-08}$, $-6.4\times 10^{-05}$). (See Fig. 8) the value of R rotates statically about one , where it is the value concerned to judge the quality of the operations.

The performance result of the LMBNN is thouhtful with the standard numerical result presented from the Runge-Kutta technique along with the input error dynamics between 0 and 1 with step-size 0.001 has come (See Fig. 9). The maximum error achieved for a data operations are less than ($1\times 10^{-06}$, $1\times 10^{-05}$, $2\times 10^{-03}$, $4\times 10^{-05}$, $5\times 10^{-06}$, $4\times 10^{-02}$) respectively.

Moreover, the LMBNN results are clarified for the ($f(\eta )$, $f'(\eta )$) for different scenarios of squeezing flow model which is shown (See Figs. 10a, c, e, 11a, c, e) respectively. And it corresponds with the given results from the Runge-Kutta numerical solution in impact scenarios and cases. In (Figs. 10a, c, e, 11a) offer the effect of the positive and negative cases of the Reynolds number *Re* on the each of the profiles $f(\eta )$, $f'(\eta )$ , and since it is clear that an increase in the value of *Re* leads to an increase in the value of profiles. While, in (Fig. 11c, e), we note that an increase in the value of the volume flow rate *Q* leads to a decrease in the value of both profiles $f(\eta )$, $f'(\eta )$. But in the Fig. 10c the velocity profile is increasing function of *Re* for $\eta$ less than 0.4 and reverse trend is observed when $\eta$ is greater than 0.4. The absolute error for different scenarios is calculated from standard solutions (See Figs. 10b, d, f, 11b, d, f), respectively. indicate that AE is about ($10^{-07}$ to $10^{-06}$ ,$10^{-07}$ to $10^{-03}$, $10^{-07}$ to $10^{-03}$, $10^{-07}$ to $10^{-05}$, $10^{-08}$ to $10^{-04}$, $10^{-07}$ to $10^{-03}$) for scenarios respectively.

Finally, the solution processes appeared while, running LMBNN, such as MSE, performance, gradient, Mu, epochs, and the time each of the three cases is listed in Table 2. The performance of LMBNN in Table 2 is around ($10^{-14}$ to $10^{-13}$, $10^{-12}$ to $10^{-06}$, $10^{-12}$ to $10^{-07}$, $10^{-12}$ to $10^{-07}$, $10^{-13}$ to $10^{-07}$, $10^{-12}$ to $10^{-06}$) respectively. These graphical and tables results presented above discern the accuracy of using LMBNN computing to solve the variants of USF-CPP.

**Table 2:** Numerical results of LMBNN for USF-CPP.

Scenarios(s)	Cases	MSE			Performance	Grad	Mu	Epochs	Time
		Training	Validation	Testing					
(1)	1	1.06045E$-$13	1.54831E$-$13	1.38605E$-$13	1.06E$-$13	1.07E$-$08	1.00E$-$14	84	0
	2	8.50907E$-$14	1.29554E$-$13	1.54468E$-$13	8.51E$-$14	4.96E$-$09	1.00E$-$13	408	0
	3	3.33939E$-$13	4.24591E$-$13	4.28304E$-$13	3.34E$-$13	9.96E$-$08	1.00E$-$14	166	0
(2)	1	1.99739E$-$6	2.04193E$-$6	4.06458E$-$6	1.91E$-$06	4.31E$-$06	1.00E$-$07	10	0
	2	3.47887E$-$12	5.58271E$-$12	6.84800E$-$12	3.48E$-$12	9.72E$-$08	1.00E$-$14	109	0
	3	6.58822E$-$12	7.34594E$-$12	8.73345E$-$12	6.19E$-$12	1.48E$-$06	1.00E$-$13	123	0
(3)	1	2.42512E$-$12	1.72015E$-$12	2.91435E$-$12	2.43E$-$12	9.98E$-$08	1.00E$-$13	148	0
	2	7.95368E$-$7	8.44452E$-$7	9.00182E$-$7	6.40E$-$07	2.66E$-$05	1.00E$-$10	10	0
	3	7.89099E$-$7	2.29014E$-$6	1.2583E$-$6	7.24E$-$07	2.19E$-$06	1.00E$-$08	11	0
(4)	1	2.41948E$-$11	2.25057E$-$11	2.64830E$-$11	1.68E$-$11	2.12E$-$06	1.00E$-$13	96	0
	2	5.54018E$-$11	7.36111E$-$11	8.01857E$-$11	5.54E$-$11	5.48E$-$08	1.00E$-$11	325	0
	3	4.09159E$-$12	5.85578E$-$12	6.80116E$-$12	4.09E$-$12	9.84E$-$08	1.00E$-$14	117	0
(5)	1	1.24770E$-$13	2.09315E$-$13	1.34424E$-$13	1.25E$-$13	9.65E$-$08	1.00E$-$15	92	0
	2	3.37253E$-$13	3.43354E$-$13	3.72923E$-$13	3.37E$-$13	9.98E$-$08	1.00E$-$14	214	0
	3	8.63517E$-$7	8.14574EE-7	1.11317E$-$6	6.65E$-$07	2.67E$-$06	1.00E$-$10	9	0
(6)	1	4.33460E$-$6	2.38151E$-$6	4.24972E$-$6	1.75E$-$06	0.000114	1.00E$-$09	9	0
	2	4.43402E$-$6	2.65687E$-$6	2.54074E$-$6	1.97E$-$06	9.11E$-$06	1.00E$-$07	9	0
	3	5.22981E$-$12	9.56182E$-$12	8.38351E$-$12	5.20E$-$12	3.10E$-$07	1.00E$-$13	295	0

## Conclusions

In this paper, the intelligent computing paradigm of Levenberg-Marquard backpropagation neural networks (LMBNN) offered a numerical solution of USF-CPP by simplified the system into an equivalent nonlinear ordinary differential equation with suitable transformation. The Runge-Kutta method is implemented for the USF-CPP dataset by variation of Reynolds number and volume flow rate. The $70\%$, $15\%$, and $15\%$ of points are determined for training, testing, and validation for various scenarios of LMBNN. The best agreement of both proposed and reference results along with the level is $10^{-06}$ to $10^{-14}$. Also, The velocity profile $f'(\eta )$ is directly proportional to the increase of Reynolds number *Re* and inversely proportional to the volume flow rate *Q*. Moreover, verifying the scheme accuracy results is achieved through graphs and tables illustrations such as mean square error, state transition dynamics, analysis of error histograms, and regression.

In the future, it will introduce mechanics through new platforms based on artificial intelligence to provide more accurate and efficient results^33–36^.

## Abbreviations

NN: Neural network
LMB: Levenberg–Marquard backpropagation
$$\rho $$: Fluid density
$$\mu $$: Dynamic viscosity
*w*: Axial velocities
$2\ell (t)$: Distance between the plates at any time t
Q: The volume flow rate
*p*: The pressure
$$\eta $$: Dimensionless variable
*u*: Radial velocities
$\nu$: The velocity of the circular plates
Re: Reynolds number

## References

[1] Stefan J (1875). Versuche über die scheinbare Adhäsion. Annalen der Physik.

[2] Ahmed Z (2021). Squeezing flow of Carbon nanotubes-based nanofluid in channel considering temperature-dependent viscosity: A numerical approach. Arab. J. Sci. Eng..

[3] Çelik İ, Öztürk HK (2021). Heat transfer and velocity in the squeezing flow between two parallel disks by Gegenbauer Wavelet Collocation method. Arch. Appl. Mech..

[4] Sobamowo G (2019). Unsteady Casson nanofluid squeezing flow between two parallel plates embedded in a porous medium under the influence of magnetic field. Open J. Math. Sci..

[5] Çelik İ (2019). Squeezing flow of nanofluids of Cu-water and kerosene between two parallel plates by Gegenbauer Wavelet Collocation method. Eng. Comput..

[6] Muhammad N, Nadeem S, Mustafa T (2017). Squeezed flow of a nanofluid with Cattaneo-Christov heat and mass fluxes. Res. Phys..

[7] Usman M (2020). Novel modification in wavelets method to analyze unsteady flow of nanofluid between two infinitely parallel plates. Chin. J. Phys..

[8] Thumma T, Magagula VM (2020). Transient electromagnetohydrodynamic radiative squeezing flow between two parallel Riga plates using a spectral local linearization approach. Heat Transf. Asian Res..

[9] Usman M (2020). Novel operational matrices-based method for solving fractional-order delay differential equations via shifted Gegenbauer polynomials. Appl. Math. Comput..

[10] Atlas M, Hussain S, Sagheer M (2019). Entropy generation and unsteady Casson fluid flow squeezing between two parallel plates subject to Cattaneo-Christov heat and mass flux. Eur. Phys. J. Plus.

[11] Rout BC, Mishra SR, Thumma T (2019). Effect of viscous dissipation on Cu-water and Cu-kerosene nanofluids of axisymmetric radiative squeezing flow. Heat Transf. Asian Res..

[12] Al-Saif, A. S. J. A. & Jasim, A. M. A novel algorithm for studying the effects of squeezing flow of a Casson fluid between parallel plates on magnetic field. *J. Appl. Math.* **2019**, (2019).

[13] Bhaskar K, Sharma K (2020). Unsteady MHD squeezing viscous Casson fluid flow in upright channel with cross-diffusion and thermal radiactive effects. Indian J. Phys..

[14] Nisar, K. S. *et al.* Numerical simulation of mixed convection squeezing flow of a hybrid nanofluid containing magnetized ferroparticles in $$50\%:$$$$50\%$$ of ethylene glycol–water mixture base fluids between two disks with the presence of a non-linear thermal radiation heat flux. *Front. Chem.* **8**, (2020). 10.3389/fchem.2020.00792 PMC7538667 33173761

[15] Waseem W (2020). Investigation of singular ordinary differential equations by a neuroevolutionary approach. PLoS ONE.

[16] Waseem W (2020). A study of changes in temperature profile of porous fin model using cuckoo search algorithm. Alex. Eng. J..

[17] Bukhari A (2020). Design of a hybrid NAR-RBFs neural network for nonlinear dusty plasma system. Alex. Eng. J..

[18] Ahmad I (2019). Novel applications of intelligent computing paradigms for the analysis of nonlinear reactive transport model of the fluid in soft tissues and microvessels. Neural Comput. Appl..

[19] Ahmad I (2020). Integrated neuro-evolution-based computing solver for dynamics of nonlinear corneal shape model numerically. Neural Comput. Appl..

[20] Bukhari AH (2020). Fractional neuro-sequential ARFIMA-LSTM for financial market forecasting. IEEE Access.

[21] Ara A (2018). Wavelets optimization method for evaluation of fractional partial differential equations: An application to financial modelling. Adv. Differ. Equ..

[22] Khan I (2020). Design of neural network with Levenberg-Marquardt and Bayesian regularization backpropagation for solving pantograph delay differential equations. IEEE Access.

[23] Sabir Z (2020). Integrated intelligent computing paradigm for nonlinear multi-singular third-order Emden-Fowler equation. Neural Comput. Appl..

[24] Sabir Z (2020). Design of neuro-swarming-based heuristics to solve the third-order nonlinear multi-singular Emden-Fowler equation. Eur. Phys. J. Plus.

[25] Raja MAZ (2018). Design of artificial neural network models optimized with sequential quadratic programming to study the dynamics of nonlinear Troesch is problem arising in plasma physics. Neural Comput. Appl..

[26] Ahmad I (2017). Neural network methods to solve the Lane-Emden type equations arising in thermodynamic studies of the spherical gas cloud model. Neural Comput. Appl..

[27] Raja MAZ (2019). Integrated intelligent computing for heat transfer and thermal radiation-based two-phase MHD nanofluid flow model. Neural Comput. Appl..

[28] Akbar S (2017). Design of bio-inspired heuristic techniques hybridized with sequential quadratic programming for joint parameters estimation of electromagnetic plane waves. Wirel. Pers. Commun..

[29] Faisal F, Shoaib M, Raja MAZ (2020). A new heuristic computational solver for nonlinear singular Thomas-Fermi system using evolutionary optimized cubic splines. Eur. Phys. J. Plus.

[30] Shoaib M (2021). A stochastic numerical analysis based on hybrid NAR-RBFs networks nonlinear SITR model for novel COVID-19 dynamics. Comput. Methods Progr. Biomed..

[31] Cheema TN (2020). Intelligent computing with Levenberg-Marquardt artificial neural networks for nonlinear system of COVID-19 epidemic model for future generation disease control. Eur. Phys. J. Plus.

[32] Rashidi MM, Siddiqui AM, Rastegari MT (2012). Analytical solution of squeezing flow between two circular plates. Int. J. Comput. Methods Eng. Sci. Mech..

[33] Shoaib, M. *et al.* A novel design of three-dimensional MHD flow of second-grade fluid past a porous plate. *Math. Probl. Eng.* **2019**, (2019).

[34] Imran A (2019). MHD and heat transfer analyses of a fluid flow through scraped surface heat exchanger by analytical solver. AIP Adv..

[35] Ahmad I (2021). A novel application of Lobatto IIIA solver for numerical treatment of mixed convection nanofluidic model. Sci. Rep..

[36] Shoaib M, Raja MAZ, Sabir MT, Islam S, Shah Z, Kumam P, Alrabaiah H (2020). Numerical investigation for rotating flow of MHD hybrid nanofluid with thermal radiation over a stretching sheet. Sci. Rep..

